# Identifying latent classes of longitudinal change in picture naming in a population-based sample

**DOI:** 10.1007/s40520-025-03169-3

**Published:** 2025-08-29

**Authors:** Deborah Finkel, Ying Liu, Margaret Gatz, Stefan Schneider, Raymond Hernandez, Bart Orriens, Arie Kapteyn

**Affiliations:** 1 Center for Economic and Social Research, University of Southern California, Los Angeles, CA USA; 2 Institute for Gerontology, School of Health and Welfare, Jönköping University, Jönköping, Sweden; 3 Department of Psychology, University of Southern California, Los Angeles, CA USA; 4 Leonard Davis School of Gerontology, University of Southern California, Los Angeles, CA USA

**Keywords:** Vocabulary, Latent growth curves, Latent class models, Adulthood, Aging, Longitudinal, Cognitive impairment

## Abstract

Although cognitive changes may not become apparent until after age 65, many factors associated with late-life decline are already well-established in midlife. In particular, deficits in picture naming have been associated with early and accelerated cognitive change. A measure of picture vocabulary, requiring participants to name drawings of objects, was collected in 4 waves (each separated by 2 years) of the Understanding America Study, a nationally representative internet panel (mean follow-up = 5.60 years). Participants were 5005 adults ranging in age from 18 to 98 at intake (mean = 48.81); 58% women. Growth mixture models were used to identify latent class structure in age-based quadratic growth models (centered at median age of 53). The best-fitting model identified 3 classes: high intercept and scores increase with age (60% of the sample), medium intercept and scores increase with age (37%), low intercept and no change with age (3%). Analyses of variance indicated that the class for which picture naming did not change with age had a significantly elevated probability of cognitive impairment. Multinomial logistic regression indicated that probability of cognitive impairment contributed to estimation of class membership even in the context of related demographic and cognitive variables. Tasks like Picture Vocabulary may be useful early indicators of onset of cognitive impairment.

**Supplementary Information:**

The online version contains supplementary material available at 10.1007/s40520-025-03169-3.

## Introduction

Picture naming is a standard measure of access to semantic knowledge and performance generally increases in young adulthood, followed by declines in later adulthood [1]. In a comparison of four measures of vocabulary, picture naming showed the strongest growth in early adulthood and the strongest decline in late adulthood [2]. Moreover, whereas all age differences in the other three vocabulary measures (synonyms, antonyms, WAIS vocabulary) were accounted for by measures of reasoning, spatial visualization, memory, and processing speed, the same was not true for picture naming. Several mechanisms for the decline in picture naming have been proposed, including inability to inhibit irrelevant information [1], slowing in rate of information processing [3, 4], and changes in spreading of activation [2, 4] – all mechanisms that may be accelerated with dementia.

In fact, unlike measures of general knowledge, deficits in picture naming have been associated with early and accelerated cognitive decline [5, 6]. Studies typically focus on identifying adults with mild cognitive impairment (MCI) or Alzheimer’s disease (AD) and comparing groups on a variety of potential indicators of cognitive deficits. Cross-sectional studies show that performance on a picture naming task differentiates control participants from participants with MCI, mild AD, and more severe AD (e.g [6–9], although some studies suggest that recall of proper names (memory for persons) is a more sensitive measure [5, 6]. Longitudinal studies measuring cognitive function at baseline and then following up a sample with AD or MCI report that picture naming declined significantly after only two years in a sample with AD [10]; however, picture naming at baseline did not differentiate between MCI groups that did or did not convert to AD after 2 years [9].

Researchers are beginning to focus on cognitive decline over time rather than cognitive performance at baseline as an early indicator of AD [11, 12], although with mixed results. For example, a study that followed adults with MCI for up to 6 years found that those who progressed to AD had a lower mean level of performance on picture naming, but no difference in rate of change over time [13]. Yet, over a similar follow-up period, Bennet and colleagues found significantly faster declines in multiple cognitive domains (episodic memory, semantic memory, and perceptual speed) in persons with MCI as compared with person without MCI [14]. Chen and colleagues [11] assessed change in cognitive function over 4 years in a community sample of adults over aged 65. Participants who developed AD during the study declined significantly faster on cognitive measures, including picture naming, than those who did not develop AD.

The current analysis used a nationally representative sample of adults and measured cognitive function over time and thus builds on previous research in two important ways. First, previous investigations of picture naming as an early indicator of accelerated cognitive change typically focused on small samples with identified MCI or AD. In contrast, the current analysis has sufficient sample size to detect possible early changes in picture naming in a representative sample of adults. In other words, instead of using experimenter-defined (a priori) groups of MCI vs. AD, models used an empirical approach to identify latent groups *post hoc* [15] that could then be compared on variables of interest, including demographic variables, related measures of cognitive function, and probability of cognitive impairment in adulthood. Second, many studies have relied on a single measurement of picture naming to identify accelerated cognitive change, whereas, in the current analysis longitudinal measurement of picture naming will support investigation of age-related trajectories in performance. Moreover, use of the full range of adulthood allows for increased accuracy in identifying a latent class structure based on trajectories of change in picture naming over age, avoiding possible observational window bias [16] that could result with a more limited age range. Latent growth curve models were applied to data from 5005 adults aged 18 to 98 who participated in 3 or 4 waves of measurement (up to 6 years of follow-up) as part of the Understanding America Study [17]. We predict that picture naming performance will generally increase from young adulthood to mid-adulthood, and that any latent class identified by deficits in picture naming will also be associated with lower education level, poorer lexical memory, and higher probability of cognitive impairment.

## Method

### Participants

The Understanding America Study (UAS) is a nationally representative internet-based panel study randomly recruited through address-based probability sampling starting in 2014 [17]. Over 14,000 U.S. residents aged 18 and older have been recruited on a rolling basis to date; they are provided with a tablet with internet access if they do not have prior access to the Internet. Panel members are invited to surveys once or twice monthly, which take an average of 15 min and cover a wide range of topics such as health, financial situation, attitudes and behaviors. Cognitive surveys repeat every 2 years. Participants recruited between 2014 and 2019 (*N* = 8154) had the opportunity to complete 3 or more waves of Picture Vocabulary testing required for the current analyses; 7730 (94.8%) of those participants were native English language speakers. The sample for the current analyses includes the 5005 native English language speakers (57.9%) who completed 3 or 4 waves of testing. Mean number of waves of participation was 3.63 (SD = 0.48) and the mean gap between waves was 2.13 years (SD = 0.21). Mean length of follow-up was 5.60 years (SD = 1.00). The sample was 75.0% non-Hispanic white, 9.8% Hispanic, 8.5% Black, 1.2% Native American, 1.2% Asian, 0.1% Pacific Islander, and 4.3% mixed race. Age at baseline ranged from 18 to 98 and generally followed a normal distribution with mean age 48.81 years (SD = 15.34).

### Measures

#### Picture vocabulary (PV)

A version of the Woodcock Johnson Picture Vocabulary test [18–20] was used to assess picture naming. Participants were presented with a picture and responded by typing the name of the object into their device (computer/laptop, tablet, or smart phone). Misspelling was permitted following the guideline that oral pronunciation of the misspelled word should correspond to the correct answer. Two forms of the list were used to mitigate the practice effect: form A included 15 items and form B included 4 items that overlapped with form A and 12 new items. Items on each form increased in difficulty from the beginning to the end of the test, and Form A and form B alternated at each wave. Scores of the two alternate forms were calibrated using the Item Response Theory [21] to ensure their comparability and also to account for the potential practice effect due to the overlapping items across forms; details can be found in the Supplemental Methods. The Supplemental Method includes mean scores with and without adjustment for practice and indicates very little practice effect.

#### Probability of cognitive impairment (PCI)

Researchers at the UAS applied the Langa-Weir criteria for dementia and MCI [24] to the on-line data collected by the UAS. Langa et al. [24] derived the Langa-Weir criteria for dementia using questions administered by interview which were then validated against clinical diagnoses based on complete clinical workups. The UAS added a separate calibration sample to determine corresponding cutoffs for the on-line version of the questions through administering the same items both by interview and on-line with a short time lag [25]. Following Langa-Weir, questions included immediate and delayed recall of 10 words and serial sevens (sequentially subtracting 7 from the previous number, starting at 100), both from the Telephone Interview for Cognitive Status (TICS; [26], and instrumental activities of daily living (difficulties using a telephone, taking medication, handling money, shopping, and preparing meals). Using cutoffs for cognitive impairment corresponding to Langa-Weir, items were combined to create an index of Probability of Cognitive impairment (or PCI) for participants aged 50 years and older (*N* = 1885); higher scores indicate a higher probability of cognitive impairment. In addition, scores on the four measures contributing to the PCI were summed to create a PCI Sum Score. The PCI Sum Score was available for everyone with data on all 4 components. Each individual component (including IADL) was scored so that higher scores indicated better performance and higher scores on the PCI Sum Score indicated better performance.

#### Cognitive measures

Two other measures from the Woodcock-Johnson cognitive tests were also collected on a biennial schedule: Number Series, a measure of quantitative reasoning, and Verbal Analogies, a measure of lexical knowledge [19]. Education was assessed in a core survey just prior to the first wave of PV assessment using a 16-point scale ranging from 1 (less than first grade) to 16 (doctoral degree). Between waves 3 and 4, a new biennial survey was added comprising a measure of Figure Identification, which assesses processing speed.

**Fig. 1:**
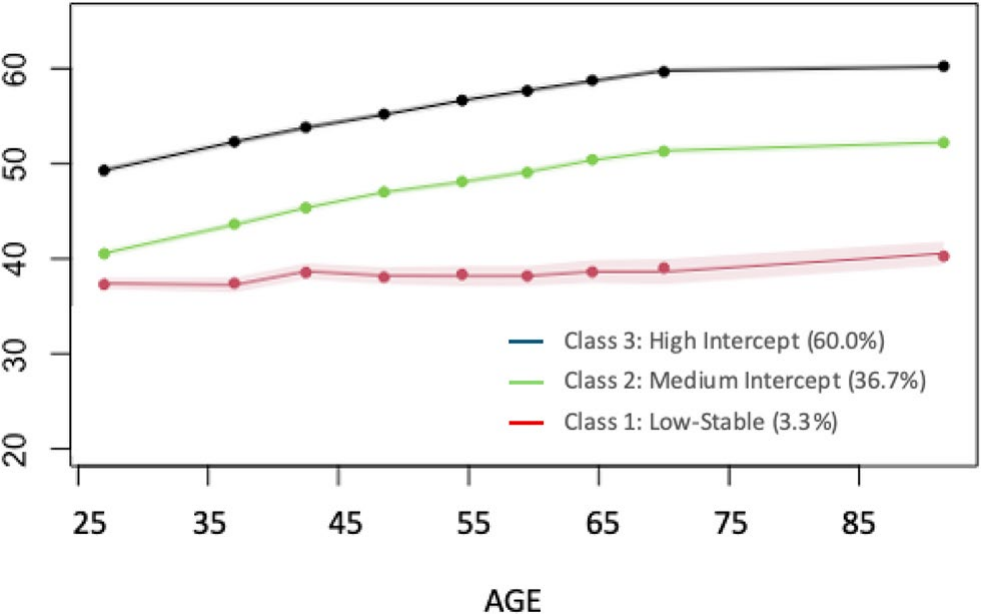
Estimated longitudinal trajectories for the classes identified in the 3-class model; shaded areas indicate confidence intervals. Percentage of the sample falling in each class is indicated in parentheses

### Additional measures

Two measures of subjective memory concern were collected in a core survey just prior to the first wave of PV: How is your memory (rated from 1 = excellent to 5 = poor) and has your memory gotten worse (rated from 1 = better to 3 = worse). This survey also asked participants to rate their eyesight for seeing things up close, like reading, and a measure of self-rated health (SRH); both were assessed on a scale from 1 (excellent) to poor (5).

### Statistical approach

Statistical approach involved 3 steps. First, growth mixture models (GMM) were used to identify latent classes of longitudinal trajectories in the sample. GMM are a multilevel modeling technique, similar to SEM and hierarchical linear growth modeling, that allows for the empirical identification of subgroups with homogenous trajectories from a large, heterogeneous sample with longitudinal data [15]. Given the age range in the sample, the current analyses used an age-based growth curve model to estimate the trajectories of change in PV with age, centered at the median age of 53, corrected for sex. Preliminary analyses indicated that a quadratic model (AIC = 112681.2) provided a better fit to longitudinal change with age than a two-slope spline model (AIC = 112700.4); therefore, the quadratic model was used in the GMM. For each class, intercept, linear change, and quadratic change with age were estimated, as well as the proportion of the sample in that class. Selection of the best model involves multiple steps including model convergence, examining information criteria (AIC, BIC, and sample-sized adjusted BIC), likelihood ratio tests of nested models, examining entropy, and substantive considerations [27]. GMM were fit using the hlme (heterogenous linear mixed models) function, which is part of the lcmm R package [28] in R version 4.3.2 [29].

Second, analysis of variance and chi-square tests were used to examine differences between classes on demographic and cognitive measures. Third, multinomial logistic regression was used to identify the variables associated with class membership, in the context of other related variables, with the largest class used as the reference class. All variables were entered into the regression simultaneously, and the global null hypothesis that none of variables were significant was tested using the likelihood ratio chi-square test. The significance of individual variables was tested using the Wald chi-square test, and odds ratios and their 95% confidence intervals were examined. Class comparisons and logistic regression were conducted using SAS version 9.4 [30].

## Results

### Descriptive statistics

Descriptive statistics for all variables included in the current analyses are presented in Supplemental Table 1, along with ranges in values. Sample sizes varied as a result of missing value or testing schedules. A total of 7730 native English speakers joined the UAS study in time to complete 3 or 4 waves of participation in the picture vocabulary task prior to the current analyses. Supplemental Table 2 examines the differences between the sample who completed 3 or 4 waves (*N* = 5005) and the 2725 who have not yet completed at least 3 waves. Note that the UAS continues to contact individuals who may be delayed in completing their monthly survey invitations; therefore, these data may be completed at a later time. Compared to the non-completers, the current sample had a significantly higher mean age (48.81 vs. 44.38). As a result of this age difference the current sample also had significantly more education (11.17 vs. 11.00), lower SRH (2.55 vs. 2.63), and higher PV scores (51.13 vs. 48.81). The largest difference between completers and non-completers was in race-ethnicity: the sample with 3 or 4 waves of participation was 25.00% non-White, whereas the sample with fewer than 3 waves was 44.38% non-White. Comparison of the sample for the current analyses to the full UAS sample is provided in Supplemental Table 3. The current sample is similar to the full UAS sample with regard to sex, age, and education and thus equally representative of the general population on these variables. However, the current sample includes fewer non-White participants (25%) than expected compared to the full UAS sample (39%).

**Table 1:** Model fitting results

Model	*N* parameters	Log Likelihood	AIC	BIC	SABIC	Entropy
1 class	11	-56259.57	112541.14	112612.84	112577.9	1
2 classes	15	-56178.30*	112386.61	112484.38	112436.7	0.64
3 classes	19	-56159.54*	112357.09	112480.93	112420.6	0.56
4 classes	23	-56152.14	112350.28	112500.20	112427.1	0.42

Note: AIC = Akaike Information Criterion, BIC = Bayesian Information Criterion, SABIC = sample-size adjusted BIC

* Model fit differs significantly from previous model at *p* < .01

**Table 2:** Comparison of differences across the 3 latent classes: means (SD)

Variable	Class 1 Low-Stable *N* = 167	Class 2 Medium Intercept *N* = 1838	Class 3 High Intercept *N* = 3000	Statistical Tests of Class Differences
Percent Female	58.68	59.58	56.83	c^2^ (df = 2) = 3.56, *p* > .15
Percent non-White	59.28	39.06	15.10	c^2^ (df = 2) = 450.55, *p* < .01
Wave 1 Age	54.26 (13.17)	47.45 (15.65)	49.35 (15.16)	F(2,5002) = 19.77, *p* < .01
Number of waves	3.61 (0.49)	3.59 (0.49)	3.65 (0.48)	F(2,5002) = 7.56, *p* < .01
Wave 1 Device				c^2^ (df = 4) = 120.86, *p* < .01
Computer	40.72%	52.72%	65.87%	
Smartphone	37.72%	34.33%	22.63%	
Tablet	21.56%	12.95%	11.50%	
SRH^a^	3.13 (1.12)	2.66 (1.04)	2.45 (0.95)	F(2,4987) = 57.18, *p* < .01
Wave 1 Education	9.21 (2.24)	10.47 (2.11)	11.70 (2.05)	F(2,5002) = 275.66, *p* < .01
Vision^a^	3.12 (1.23)	2.58 (1.20)	2.32 (1.12)	F(2,4987) = 58.68, *p* < .01
Rate Memory^b^	2.77 (0.92)	2.48 (0.94)	2.33 (0.89)	F(2,4731) = 28.40, *p* < .01
Memory Change^b^	1.99 (0.44)	2.04 (0.40)	2.08 (0.37)	F(2,4731) = 9.58, *p* < .01
Number Series	40.10 (9.31)	47.27 (8.52)	53.66 (8.23)	F(2,5002) = 474.66, *p* < .01
Verbal Analogies	37.13 (8.77)	46.71 (8.81)	53.80 (6.74)	F(2,5002) = 760.87, *p* < .01
Figure Identification	12.04 (4.92)	16.26 (5.67)	18.35 (5.65)	F(2,5002) = 149.75, *p* < .01
PCI^c^	0.16 (0.17)	0.09 (0.12)	0.04 (0.08)	F(2,1882) = 74.94, *p* < .01
PCI Sum Score^d^	15.15 (5.13)	17.72 (4.34)	20.17 (3.83)	F(2,3538) = 186.86, *p* < .01
Immediate Recall^d^	3.37 (1.80)	4.25 (1.19)	4.59 (0.85)	F(2,3538) = 95.32, *p* < .01
Delayed Recall^d^	4.18 (2.16)	4.87 (1.91)	5.82 (1.76)	F(2,3538) = 129.49, *p* < .01
Serial Sevens^d^	2.89 (2.36)	3.79 (2.12)	4.86 (1.99)	F(2,3538) = 132.61, *p* < .01
IADL^d^	4.74 (0.69)	4.82 (0.60)	4.89 (0.47)	F(2,3538) = 11.56, *p* < .01

^a^ Class Ns = 166, 2989, 1835

^b^ Class Ns = 158, 2848, 1728

^c^ Class Ns = 61, 1218, 606

^c^ Class Ns = 84, 2236, 1221

**Table 3:** Multinomial logistic regression to predict membership in the 3 latent classes. Reference = High intercept class

Variable	Estimate (SE)	Significance	Odds Ratio	95% C.I.
Age				
Low-Stable class	0.01 (0.01)	0.30	1.01	0.99, 1.03
Medium Intercept class	-0.02 (0.00)	0.00	0.98	0.96, 0.99
Sex (0 = female, 1 = male)				
Low-Stable class	0.58 (0.29)	0.04	1.78	1.01, 3.12
Medium Intercept class	0.23 (0.09)	0.01	1.26	1.05, 1.50
Race (0 = non-White, 1 = White)				
Low-Stable class	-1.77 (0.30)	0.00	0.17	0.10, 0.31
Medium Intercept class	-0.90 (0.10)	0.00	0.41	0.33, 0.50
Education				
Low-Stable class	-0.20 (0.07)	0.01	0.82	0.72, 0.94
Medium Intercept class	-0.11 (0.02)	0.00	0.89	0.85, 0.93
SRH (1 = excellent; 5 = poor)				
Low-Stable class	0.17 (0.14)	0.23	1.19	0.90, 1.57
Medium Intercept class	-0.05 (0.05)	0.27	0.95	0.86, 1.04
Vision (1 = excellent; 5 = poor)				
Low-Stable class	0.14 (0.12)	0.24	1.16	0.91, 1.47
Medium Intercept class	0.13 (0.04)	0.00	1.14	1.06, 1.23
Number of Waves				
Low-Stable class	-0.15 (0.29	0.60	0.86	0.49, 1.52
Medium Intercept class	-0.02 (0.10)	0.84	0.98	0.81, 1.18
Self-rated Memory				
Low-Stable class	0.20 (0.16)	0.22	1.22	0.89, 1.66
Medium Intercept class	0.06 (0.05)	0.26	1.06	0.96, 1.18
Self-rated Memory Change				
Low-Stable class	-0.28 (0.36)	0.44	0.75	0.37, 1.54
Medium Intercept class	-0.10 (0.12)	0.39	0.90	0.71, 1.15
Device (1 = computer, 2 = tablet/cell)				
Low-Stable class	0.23 (0.28)	0.42	1.26	0.72, 2.20
Medium Intercept class	0.13 (0.09)	0.16	1.13	0.95, 1.35
Number Series				
Low-Stable class	-0.03 (0.02)	0.14	0.97	0.93, 1.01
Medium Intercept class	-0.03 (0.01)	0.00	0.97	0.96, 0.98
Verbal Analogies				
Low-Stable class	-0.17 (0.02)	0.00	0.85	0.82, 0.88
Medium Intercept class	-0.07 (0.01)	0.00	0.93	0.92, 0.95
Figure Identification				
Low-Stable class	-0.14 (0.03)	0.00	0.87	0.81, 0.92
Medium Intercept class	-0.06 (0.01)	0.00	0.95	0.93, 0.96
PCI Sum Score				
Low-Stable class	-0.14 (0.03)	0.00	0.87	0.82, 0.93
Medium Intercept class	-0.08 (0.01)	0.00	0.92	0.90, 0.94

### Growth mixture modeling

Results of fitting four growth mixture models to the longitudinal PV data are presented in Table 1 and parameter estimates from the four models are presented in Supplemental Table 2. The first model fit the age-based quadratic growth curve model to the whole sample, the longitudinal trajectory estimated from this model is presented in Supplemental Fig. 1. The second model estimated 2 classes and fit significantly better than the first model: LRT = 81.27, df = 4, *p* < .01. Class 1 from this model, including 8.5% of the sample, had a lower intercept and less change with age than Class 2 (91.5% of the sample), trajectories estimated from the 2-class model are presented in Supplemental Figure F1. The third model estimated 3 classes and fit significantly better than the 2-class model: LRT = 18.76, df = 4, *p* < .01. The fourth model estimated 4 classes and did not improve model fit over the 3-class model: LRT = 7.40, df = 4, *p* > .10). The BIC and SABIC model fit statistics were also minimized for the 3-class model; therefore, the 3-class model was selected as the best fitting model. Longitudinal trajectories estimated for the 3 classes are presented in Fig. 1; raw longitudinal trajectories are presented in Supplemental Fig. 2. Class 1 (3.34% of the sample) – “Low-Stable” – had the lowest intercept and neither linear nor quadratic change parameters differed significantly from zero, indicating no change in mean score on PV with age. Class 2 (36.72%) – “Medium Intercept” – had an intermediate intercept and demonstrated decelerating increases in PV with age. Class 3 (59.94%) – “High Intercept” – had the highest intercept and demonstrated changes in PV with age that was parallel to the longitudinal trajectory for Class 2. Although the sample size was small for the Low-Stable Class, the fact that both 2-class and 3-class models (and even the 4-class model) identified this group increases confidence in its validity. GMM was repeated in the reduced sample with PCI Sum Score (*N* = 3541) and results were similar, with one class identified that showed little or no improvement in PV with age. As a result of small sample size in late adulthood, the estimated mean trajectories for each class did not indicate expected declines in PV in late adulthood that can be detected in the raw trajectories. Cross-sectional means at each wave, reported in Supplemental Table 5, indicate both the reduction in sample size in late adulthood, as well as the expected decline in mean PV. The table also includes mean education level for participants in each age decade, indicating highest mean education levels in the oldest ages (ages 70 and higher); differences in mean education level differed significantly across age decades (*F*(7, 4997) = 11.41, *p* < .01).

### Class comparisons

The next step of the analyses was to determine how the 3 classes identified by GMM differed on demographic variables and other cognitive variables. Results of comparing variables across the 3 classes are presented in Table 2; due to missing data, the Ns for the classes varied somewhat for some variables. Results show that the 3 classes differed significantly on every variable included, with the exception of sex. Moreover, follow-up comparisons indicated that the 3 classes differed significantly from each other for all variables with the exception of number of waves of participation (Medium Intercept Class differed from High Intercept Class) and IADL (Low-Stable Class does not differ from Medium Intercept Class). Given the large differences in sample size, comparisons across the 3 classes were repeated using the nonparametric Kruskal-Wallis test. Results were the same with one exception: follow-up testing indicated that for self-reported memory change, the Low-Stable Class did not differ significantly from the Medium Intercept Class. All 3 classes averaged about 3.6 waves of participation and the significant differences between classes arose from the minimal standard deviation resulting from the reduced range in number of waves of participation (3 or 4 waves).

Compared with the other two classes, the Low-Stable Class had the highest percentage of non-White participants (59.28%), had the highest mean age (54.26), was more likely to use a smart phone or tablet to complete the surveys, had worse SRH, had less education (mean of 9.21 is equivalent to high school), had poorer vision, rated their memory as worse, and scored lower on all cognitive measures. Importantly, participants in the Low-Stable Class had the highest probability of cognitive impairment: mean probability was 16% which was nearly twice the probability for participants in the Medium Intercept Class and 4 times the probability for participants in the High Intercept Class. Contrary to the general pattern, participants in the Low-Stable Class were least likely to report that their memory had gotten worse, and they did not differ from the Medium Intercept Class on the IADL measure: both groups had lower IADL performance than the High Intercept Class.

The Medium Intercept and High Intercept Classes both demonstrated improved PV performance with age and the High Intercept Class had a higher intercept. The High Intercept Class had the lowest percentage of non-White participants (15.10%), had an intermediate mean age (49.25), was most likely to complete the survey using a computer, had the best SRH, the highest level of education (mean of 11.70 is equivalent to 2 years of college), best self-reported vision, best self-reported memory, and scored highest on all cognitive measures.

### Multinomial logistic regression

Given the interrelationships among the demographic and cognitive variables reported in Table 2, multinomial logistic regression was used to identify the variables that were associated with class membership in the context of the other variables. Variables included in the multinomial logistic regression were age, sex, race, waves of participation, education, SRH, vision, self-rated memory, self-rated memory change, device, Number Series, Verbal Analogies, Figure Identification, and PCI Sum Score. PCI was available only for participants aged 50 years and older, had significant positive skew, and 27% of the values were less than 0.01. The PCI Sum Score was available for all participants and was normally distributed; therefore, the PCI Sum Score was used in the logistic regressions. Race was dichotomized to non-White and White, and device used to complete the survey was dichotomized to computer and tablet/smartphone. A subset of 3388 participants had data on all included variables: 77 in the Low-Stable Class, 1171 in the Medium Intercept Class, and 2140 in the High Intercept Class. Results of logistic regression to estimate membership in each class are reported in Table 3. The likelihood ratio chi-square testing the global null hypothesis was significant (1172.28, df = 28, *p* < .01).

Sex, race, education, Verbal Analogies, Figure Identification, and the PCI Sum Score contributed consistently to estimation of class membership. Compared with the High Intercept Class, individuals in the Low-Stable and Medium Intercept Classes were more likely to be men, non-White, have lower education, and lower scores on Verbal Analogies, Figure Identification, and the PCI Sum Score. For 5 of these variables, the odds ratio for the Low-Stable Class was significantly different from the odds ratio for the Medium Intercept Class; thus, compared the Medium Intercept Class, individuals in the Low-Stable Class were more likely to be non-White, have lower education, and lower scores on Verbal Analogies, Figure Identification, and the PCI Sum Score. The odds ratios for Vision and Number Series were the same or similar for the Low-Stable and Medium Intercept Classes; however, given differences in sample size the odds ratios were significant only for the Medium Intercept Class. Lower scores on Number Series and worse Vision were associated with membership in the Medium Intercept Class. Finally, younger age was modestly associated with membership in the Medium Intercept Class. SRH, waves of participation, self-rated memory, self-rated memory change, and device did not contribute significantly to estimation of class membership.

## Discussion

The goal of the current analysis was to identify distinct patterns of longitudinal change in Picture Vocabulary during adulthood and investigate possible covariates of different rates of change with age. Growth mixture modeling of longitudinal change in Picture Vocabulary across 4 waves (up to 6 years of follow-up) identified 3 classes with distinct trajectories of change with age. The largest class (59.95%) had the highest intercept of the 3 classes and demonstrated decelerating increases in Picture Vocabulary over age. The second largest class (36.72%) demonstrated a parallel trajectory of change with age, but a lower intercept in Picture Vocabulary performance. The smallest class (3.34%) demonstrated a completely different longitudinal trajectory, with the lowest intercept and no significant change in Picture Vocabulary over age. Significant differences were found between classes in demographic variables (age, sex, and race), education, physical variables (vision and self-rated health), and cognitive variables including the Probability of Cognitive Impairment. Multinomial logistic regression indicated that the PCI Sum Score contributed to the estimation of class membership for all 3 classes, even in the context of related cognitive and demographic variables.

Increases with age in vocabulary in general and Picture Vocabulary performance in particular are expected [3]. Expected declines in late adulthood were not observed in the group mean latent trends, although they were apparent in some individual trajectories and in the cross-sectional means at each wave. Limited sample sizes at later ages reduced the power of the LGCM to model the decreases in mean Picture Vocabulary noted in the raw data. Mean education level across age decades varied from 10.61 to 11.54. Although the absolute difference is small (both level 10 and 11 indicate some college education), the cultural difference is much larger. As a result of the expansion of access to college education in the twentieth century [31], the education level of the oldest adults in the sample represents a more select sample than the younger participants. Although selective survival is an issue with any longitudinal study of aging, the older adults in the current sample were likely less representative of their age peers than the younger participants with regard to education and consequently may not have demonstrated the expected larger declines in late adulthood in Picture Vocabulary.

Notably, one class identified by growth mixture modeling did not demonstrate the expected *increases* in Picture Vocabulary from early to middle adulthood. Instead, the longitudinal trajectory estimated for the Low-Stable Class was flat, with no significant changes over age. Mean education was lowest for this class and education differentiated all three classes in the multinomial regression. It is possible, then, that participants in the Low-Stable Class were not exposed over their lifespan to opportunities to acquire the more challenging items in the Picture Vocabulary task. Lower Picture Vocabulary performance has also been associated with mild cognitive impairment and transition to AD [13]. Based on national health statistics [32] and given the age range of the current sample, we would expect that approximately 2.8% of the sample has an elevated probability for early cognitive decline associated with dementia. Given that picture naming can be an indicator of cognitive impairment, we would expect to find that approximately 3% of a nationally representative sample with the age distribution of the UAS sample would demonstrate a significantly different aging trajectory in Picture Vocabulary. The Low-Stable Class, which did not show the expected increase in picture naming with age, was 3.3% of the sample. Thus, it is possible that the growth mixture model successfully identified a subgroup that had a higher probability of early-onset cognitive decline.

Class comparisons and stepwise logistic regressions provided tests of this possibility. Participants in the Low-Stable Class demonstrated significantly elevated Probability of Cognitive Impairment compared with the other two classes; in fact, their probability was 4 times the probability of the High Intercept Class. Examining the measures that contribute to the estimation of PCI indicated consistent differences across classes: the Low-Stable Class had lower scores on Serial Sevens, Immediate Recall, Delayed Recall, and IADL, the Medium Intercept Class had intermediate scores, and the High Intercept Class had the highest score on all 4 measures, and there was no indication that the relative role of the 4 component measures differed across classes. However, the classes did differ on multiple related demographic and cognitive variables; therefore, multinomial logistic regression was used to determine the association of PCI Sum Scores with class membership in the context of these variables.

Six variables consistently distinguished between the latent classes in the multinomial regression: sex, race-ethnicity, education, Verbal Analogies, Figure Identification, and PCI Sum Score. The significance of race and education could reflect a cultural bias in the Picture Vocabulary measure and/or lack of opportunity for some participants to expand their vocabulary knowledge over adulthood. Performance on Verbal Analogies incorporates several cognitive processes, including spreading activation between nodes within an associative mental network [33, 34]. Figure Identification is considered a measure of information processing speed that is fundamentally associated with age changes in cognition (e.g. [35]). Changes in both spreading of activation [3, 4] and processing speed [4, 27] have been proposed as mechanisms for age changes in picture naming. The current results provided support for these mechanisms, in contrast to the role other cognitive measures such as Number Series, a measure of reasoning that did not reliability distinguish between the latent classes (cf. [3]). Importantly, PCI Sum Score contributed significantly to the estimation of class membership for all 3 classes, even in the context of the cognitive and demographic measures, highlighting the association between picture naming and accelerated cognitive decline.

### Implications

Alzheimer’s disease and related dementias (ADRD) are predicted to increase substantially over the next few decades as the world’s populations age [36]. In the wake of failed or modestly effective drug trials [37–39], early detection of cognitive decline may be one of the most relevant aspects of treatment [40, 41]. Identification of individuals with elevated probability for accelerated cognitive decline provides opportunities for earlier intervention, such as modifying exposure to recognized risk factors [42]. Ideally, researchers can identify measures that are relatively inexpensive and easy to use and that are sensitive enough to detect early signs of cognitive decline. For example, difficulty finding words is one of the most common complaints of older adults [4]. Indeed, picture naming may be especially sensitive to cognitive loss as it taps several cognitive processes, including attention, lexical access, spatial visualization, and semantic representations [43, 44].

### Limitations

It is in the nature of the UAS study that participants choose their own testing platform (computer, tablet, smartphone) and testing environment. Smartphones and tablets may allow tasks to be completed in a wide array of environments with greater potential for distraction compared to computers (e.g., on the bus, in a waiting room for an appointment, etc.). The Low-Stable Class had the highest level of smartphone and tablet usage; however, device was not identified in the multinomial regression as associated with class membership. Although a strength of the study is the reliance on the nationally representative UAS study, the current sample differed from the full UAS demographics with regard to race-ethnicity. The current sample had fewer non-White participants than expected at baseline and a higher proportion of White than non-White participants completed the 3 or more waves of measurement required for inclusion in the analyses. Even so, the sample was 25% non-White (*N* = 1251), which provided sufficient power to detect significant race-ethnicity differences across the latent classes. Race-ethnicity was associated with class membership in the multinomial regression, as did other demographic and cognitive variables.

## Conclusions

Distinct longitudinal trajectories were identified for change with age in picture naming, including a class that did not demonstrate the expected increases with age. Change with age, or lack thereof, in cognitive measures, particularly picture naming that requires production of names of objects, may serve as an early indicator of accelerated cognitive decline. Evidence suggests that trajectories of change in cognitive measures, including picture naming, may be more informative than baseline measures [11, 13, 14]. Repeated assessment of a simple task such as Picture Vocabulary may allow researchers to identify adults whose scores do not increase as expected with age, or adults whose scores decline earlier than expected. Earlier identification of individuals at risk for accelerated cognitive decline would provide opportunities for earlier intervention, which can reduce care costs and improve quality of life [45].

## Electronic Supplementary Material

Below is the link to the electronic supplementary material.

Supplementary Material 1

## Data Availability

Quantitative data sets are available from the Understanding America Study website (http://uasdata.usc.edu). Consistent with open science practices, any individual affiliated with a research institution is able to access these data for purpose of replication or research after free registration, and provision of a signed data use agreement. The software code that supports the findings of this study is available from the corresponding author upon reasonable request.
