# Supplementary material for: Identifying latent classes of longitudinal change in picture naming in a population-based sample
Source: Aging Clin Exp Res. 2025 Aug 29;37(1):262. doi: 10.1007/s40520-025-03169-3 (PMC12397163; doi:10.1007/s40520-025-03169-3)
Supplement: Supplementary file 1 — Supplementary Material 1 [file 40520_2025_3169_MOESM1_ESM.docx]

Supplemental Materials

- Supplemental Methods: Calibration and scoring of the alternate forms of the picture vocabulary test.
- Supplemental Table 1. Descriptive statistics for full sample included in analyses.
- Supplemental Table 2. Comparison of participants who entered UAS by 2019 (N = 7730) with and without 3 or more waves of measurement on picture vocabulary.
- Supplemental Table 3. Comparison of current sample demographics to full UAS sample.
- Supplemental Table 4. Latent growth curve parameter estimates (SE) for models with 1, 2, 3, and 4 classes.
- Supplemental Table 5: Cross-sectional age differences in mean picture vocabulary scores at each wave.
- Supplemental Fig. 1. Estimated longitudinal trajectories for the classes identified in the 1-class model (C1), 2-class model (C2:1, C2:2), and 3-class model (C3:1, C3:2, C3:3).
- Supplemental Fig. 2. Raw longitudinal trajectories for Class 1 (N = 167), a random 10% of participants in Class 2 (N = 300), and a random 10% of participants in Class 3 (N = 183).

Supplemental Methods: Calibration and scoring of the alternate forms of the picture vocabulary test

The picture vocabulary test includes two parallel test forms A and B, which were administered in rotating order across waves to limit repeated exposure to the same test items over time. Test scores of both forms were derived using Item Response Theory (IRT) models. A two-parameter logistic (2PL) IRT model was used to first calibrate the characteristics of the items based on two overlapping samples of UAS respondents: 2,832 respondents who took the form A in 2016, and 2,672 who took the form B in 2018. The estimated item characteristics were then fixed and applied to derive the person-level scores in the full English-speaking sample for all waves of the UAS.

In the 2PL-IRT model, the probability of correctly solving a test item is viewed as a function of a test taker’s ability level and the difficulty and discrimination parameters of the test item. The difficulty parameter measures the ability level at which there is a 50% chance of answering the item correctly, whereas the discrimination parameter measures how sensitive this probability is to differences in the ability level. The 2PL model allows both the difficulty and discrimination parameters to differ across test items.

For each form, we first assessed the unidimensionality of the item scores, a prerequisite for IRT modeling, using confirmatory factor analysis for binary variables. Adequate data-model fit of a one-factor model supported the unidimensionality assumption. A 2PL model was estimated using marginal maximum likelihood parameter estimation to calibrate the item parameters. The calibrated parameters were then applied to derive IRT-based scaled scores for individual respondents using the Expected A Posteriori (EAP) method. The scores were converted into T-scores, where 50 is the mean and 10 is the SD of the general US population. The T-score metric has widespread use in psychological and cognitive testing and has been adapted, for example, by the Harmonized Cognitive Assessment Protocol (HCAP; (Langa et al., 2020). A score of 50 means that the person’s cognitive ability is equal to that of the average person in the general population, a score of 60 means that the person’s ability is one standard deviation above average, and a score of 40 means that the person’s ability is one standard deviation below average.

*Linking scores across forms*. When calibrating Form A, we applied sampling weights to the IRT model in the calibration sample to ensure that the item parameters appropriately reflected those in the general population. When calibrating the Form B, to allow for changes in respondents’ true scores over time, 4 items administered in Form A were re-administered together with the items of Form B. The items were chosen to span a wide range of difficulty levels and to demonstrate at least moderate ability to differentiate between individuals (as reflected in moderate to high discrimination parameters). The item parameters of the four overlapping items were prefixed at their estimated values from the Form A calibration. This method, frequently used to link multiple forms of a test and develop a common scale (Lee & Lee, 2018), ensures that the Form B items are placed on the same metric as Form A, and in turn, ensures the person-level scale scores are comparable across forms. Further details of the calibration can be found at https://uasdata.usc.edu/index.php.

*Adjustment of the potential practice effect due to the overlapping items*. One assumption of the aforementioned linking procedure is that the people’s performance on the overlapping items would not change from one calibration wave to another. Because the UAS respondent must have taken the Form A as the first wave before they were invited to the Form B as the second wave two years later, the same four overlapping items were given in all waves, and any potential practice effect on these items could bias the Form B calibration, for instance, upward shifting the scale with spuriously higher scale scores. To correct this potential bias, when deriving the scale scores for Wave 2 and later, we applied the estimated item characteristics to the correctness score of the non-overlapping items from that wave and the Wave 1 correctness score for the overlapping items. In other words, we assumed that in the follow-up waves, the participants would have performed exactly the same as the first time when they saw the overlapping items. These corrected scale scores were used in all analyses in the current study.

Picture Vocabulary scores in full UAS sample without and with adjustments for practice effects.

| Picture Vocabulary Score | N | Without Adjustment | With Adjustment |
| --- | --- | --- | --- |
| Mean Wave 1 (SD) | 14,440 | 49.45 (9.03) | 49.45 (9.03) |
| Mean Wave 2 (SD) | 8,770 | 51.96 (9.35) | 51.34 (9.25) |
| Mean Wave 3 (SD) | 5,436 | 51.64 (8.79) | 51.03 (8.72) |
| Mean Wave 4 (SD) | 3,377 | 53.19 (9.11) | 52.77 (9.04) |

Note: Sample sizes decline across waves primarily because the UAS uses ongoing sample recruitment and not all participants have been involved in the UAS long enough to be invited to subsequent waves of cognitive testing.

Supplemental Table 1. Descriptive statistics for full sample included in analyses.

| Variable | N | Descriptive | Range |
| --- | --- | --- | --- |
| Percent Female | 5005 | 57.90% |  |
| Percent non-White | 5005 | 25.0% |  |
| Wave 1 Year | 5005 |  |  |
| 2016 |  | 72.23% |  |
| 2017 |  | 8.46% |  |
| 2018 |  | 12.52% |  |
| 2019 |  | 6.79% |  |
| Mean Wave 1 Age (SD) | 5005 | 48.81 (15.34) | 18–98 |
| Mean Number of waves (SD) | 5005 | 3.63 (0.48) | 3–4 |
| Wave 1 Device  Computer  Smartphone  Tablet | 5005 | 60.20%  27.43%  12.37% |  |
| Mean SRH (SD) | 4990 | 2.55 (1.00) | 1–5 |
| Mean Wave 1 Education (SD) | 5005 | 11.17 (2.19) | 1–16 |
| Mean Vision (SD) | 4990 | 2.44 (1.16) | 1–5 |
| Mean Rate Memory (SD) | 4734 | 2.40 (0.91) | 1–5 |
| Mean Memory Change (SD) | 4734 | 2.06 (0.38) | 1–3 |
| Mean Number Series (SD) | 5005 | 50.86 (9.14) | 13.58–65.27 |
| Mean Verbal Analogies (SD) | 5005 | 50.64 (8.72) | 13.14–60.97 |
| Mean Figure Identification (SD) | 5005 | 17.38 (5.63) | 0–30 |
| Mean PCI (SD) | 1885 | 0.06 (0.10) | 0–0.92 |
| Mean PCI Sum Score (SD) | 3541 | 19.21 (4.26) | 2–30 |
| Mean Immediate Recall (SD) | 3541 | 5.45 (1.89) | 0–10 |
| Mean Delayed Recall (SD) | 3541 | 4.45 (2.12) | 0–10 |
| Mean Serial Sevens (SD) | 3541 | 4.45 (1.04) | 0–5 |
| Mean IADL (SD) | 3541 | 4.86 (0.53) | 0–5 |

Supplemental Table 2. Comparison of participants who entered UAS by 2019 (N = 7730) and do or do not have the 3 or more waves of Picture Vocabulary required to be included in the current analyses.

| Variable | N | 3 + waves  *In the current analyses* | N | < 3 waves  *Not in the current analyses* | Statistical Test of  Group Differences |
| --- | --- | --- | --- | --- | --- |
| Percent Female | 5005 | 57.90% | 2725 | 58.32% | c^2^ (df = 1) = 0.12, *p* > .70 |
| Percent non-White | 5005 | 25.00% | 2725 | 40.43% | c^2^ (df = 1) = 187.32, *p* < .01 |
| Mean Wave 1 Age (SD) | 5005 | 48.81 (15.34) | 2725 | 44.38 (17.15) | *t* (df = 7728) = 11.64, *p* < .01 |
| Mean Education (SD) | 5005 | 11.17 (2.19) | 2724 | 11.00 (2.22) | *t* (df = 7727) = 3.26, *p* < .01 |
| Mean SRH (SD) | 4990 | 2.55 (1.00) | 1790 | 2.63 (1.00) | *t* (df = 6778) = -3.01, *p* < .01 |
| Mean Picture Vocabulary at Wave 1 (SD) | 5005 | 51.13 (8.57) | 2717 | 48.81 (8.80) | *t* (df = 7722) = 11.23, *p* < .01 |

Note: Participants who entered UAS after 2019 did not have sufficient time in the study to be invited to participate in a 3rd (4 years after entry) or 4th wave (6 years after entry) of picture vocabulary testing.

Supplemental Table 3. Comparison of current sample demographics to full UAS sample (%).

| Variable | Unweighted UAS | Weighted UAS^1^ | Current Sample |
| --- | --- | --- | --- |
| *Male* | 39.25 | 48.81 | 42.10 |
| *Race-ethnicity* |  |  |  |
| White | 58.91 | 61.10 | 75.00 |
| Black | 11.75 | 12.06 | 8.50 |
| Other | 9.29 | 7.46 | 5.50 |
| Hispanic | 16.20 | 16.91 | 9.80 |
| Native American | 3.85 | 2.47 | 1.20 |
| *Age (years)* |  |  |  |
| 18–39 | 30.24 | 37.47 | 31.79 |
| 40–49 | 19.33 | 15.92 | 18.21 |
| 50–59 | 17.75 | 15.73 | 22.18 |
| 60+ | 32.68 | 30.89 | 27.82 |
| *Education* |  |  |  |
| High school or less | 20.34 | 37.75 | 23.76 |
| Some college | 33.56 | 26.45 | 38.83 |
| Bachelor or more | 46.10 | 35.80 | 37.41 |

^1^Weighted percentages using final weights match their population counterparts by construction, given the convergence of the raking/trimming algorithm, per UAS weighting procedures

Supplemental Table 4. Latent growth curve parameter estimates (SE) for models with 1, 2, 3, and 4 classes.

| Parameter | Intercept | Sex | Linear | Quadratic | Class % |
| --- | --- | --- | --- | --- | --- |
| *Model 1* |  |  |  |  |  |
| Class 1 | 51.69 (0.15) | 2.50 (0.21) | 0.20 (0.01) | -0.002 (0.0003) | 100.00 |
| *Model 2* |  |  |  |  |  |
| Class 1 | 41.67 (0.62) | 2.36 (0.20) | 0.08 (0.02) | 0.000 (0.0010)† | 8.51 |
| Class 2 | 53.46 (0.24) | 2.36 (0.20) | 0.21 (0.01) | -0.003 (0.0003) | 91.49 |
| *Model 3* |  |  |  |  |  |
| Class 1 | 37.35 (0.78) | 2.27 (0.19) | 0.04 (0.03)† | 0.001 (0.0012)† | 3.34 |
| Class 2 | 47.51 (0.52) | 2.27 (0.19) | 0.20 (0.01) | -0.003 (0.0006) | 36.72 |
| Class 3 | 55.95 (0.36) | 2.27 (0.19) | 0.20 (0.01) | -0.003 (0.0005) | 59.94 |
| *Model 4* |  |  |  |  |  |
| Class 1 | 46.38 (0.74) | 2.27 (0.19) | 0.20 (0.02) | -0.003 (0.0006) | 32.97 |
| Class 2 | 53.92 (0.77) | 2.27 (0.19) | 0.27 (0.03) | -0.003 (0.0010) | 42.22 |
| Class 3 | 57.19 (0.73) | 2.27 (0.19) | 0.12 (0.04) | -0.002 (0.0010)† | 21.88 |
| Class 4 | 35.95 (1.17) | 2.27 (0.19) | 0.09 (0.03) | -0.000 (0.0014)† | 2.94 |

† Parameter estimate was *not* significantly different from zero at *p* < .01. All other parameters were significantly different from zero at *p* < .01.

Supplemental Table 5: Cross-sectional means (SD) in picture vocabulary scores at each wave.

| Age at Wave 1 | Education | N  Waves 1–3 | Wave 1 | Wave 2 | Wave 3 | N  Wave 4 | Wave 4 |
| --- | --- | --- | --- | --- | --- | --- | --- |
| 18–29 | 10.61 (2.09) | 626 | 44.24 (7.93) | 46.21 (8.38) | 44.68 (7.87) | 325 | 46.66 (8.51) |
| 30–39 | 11.38 (2.19) | 963 | 48.08 (7.78) | 49.64 (8.20) | 48.68 (7.97) | 607 | 50.22 (8.41) |
| 40–49 | 11.34 (2.22) | 911 | 50.73 (7.67) | 52.43 (8.31) | 51.26 (7.93) | 573 | 52.77 (8.23) |
| 50–59 | 10.95 (2.09) | 1110 | 52.43 (8.04) | 53.63 (8.23) | 52.88 (7.90) | 754 | 54.25 (8.23) |
| 60–69 | 11.24 (2.16) | 943 | 55.35 (7.50) | 56.79 (7.83) | 55.54 (7.17) | 632 | 56.98 (8.07) |
| 70–79 | 11.54 (2.38) | 390 | 56.04 (7.12) | 57.43 (7.15) | 55.84 (6.67) | 226 | 56.99 (6.65) |
| 80–89 | 11.51 (2.38) | 53 | 55.43 (7.82) | 56.02 (7.86) | 53.74 (8.45) | 27 | 53.62 (10.21) |
| 90+ | 11.50 (2.74) | 9 | 54.34 (6.56) | 55.42 (9.13) | 52.85 (8.08) | 4 | 59.00 (8.54) |

Supplemental Fig. 1. Estimated longitudinal trajectories for the classes identified in the 1-class model (C1), 2-class model (C2:1, C2:2), and 3-class model (C3:1, C3:2, C3:3). Percentage of the sample falling in each class is indicated in parentheses.


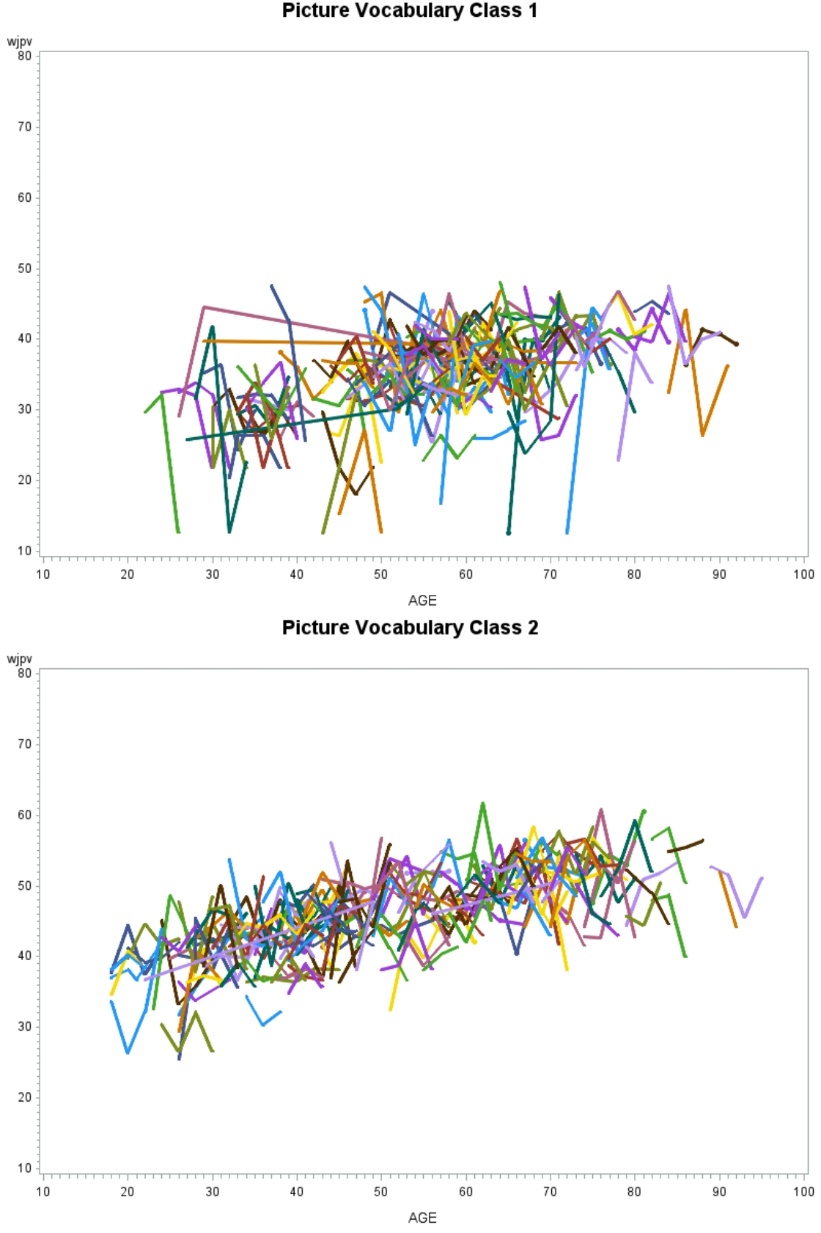


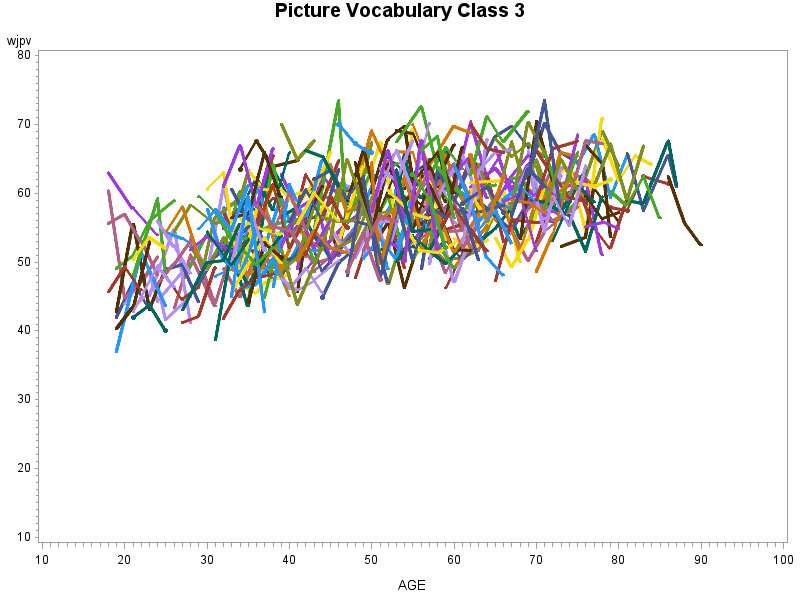


Supplemental Fig. 2. Raw longitudinal data for Class 1: Low-Stable (N = 167), a random 10% of participants in Class 2: Medium Intercept (N = 183), and a random 10% of participants in Class 3: High Intercept (N = 300).
